# Defining sepsis in the ICU: a sensitivity analysis

**DOI:** 10.1186/cc9629

**Published:** 2011-03-11

**Authors:** P Klein Klouwenberg, OL Cremer

**Affiliations:** 1 University Medical Centre Utrecht, the Netherlands

## Introduction

According to Consensus Conference [1] and PROWESS study criteria [2], the diagnosis of sepsis requires evidence of infection and the presence of a systemic inflammatory response syndrome (SIRS) that is characterized by specific physiological alterations. Although these criteria are widely accepted in clinical practice and research, they have been criticized for being nonspecific and nonrobust in both clinical practice and clinical research settings [3]. With regard to these issues, it remains unknown to what extent differences in the frequency (every minute vs. hourly), timing (SIRS criteria transiently present at any time point in the last 24 hours vs. simultaneously present during a longer period) and method (automated vs. manual) of data capture may affect the diagnosis of sepsis. In this study we aimed to quantify the effect of minor variations in the definition of SIRS on the apparent incidence of sepsis.

## Methods

We performed an observational study in consecutive patients admitted to a large tertiary ICU in The Netherlands between January 2009 and October 2010. Patients following elective surgery who had an uncomplicated stay <96 hours were excluded from analysis. We collected data on SIRS criteria and information on infectious status during the first 24 hours of admission.

## Results

In total 1,216 patients met the inclusion criteria. The incidence of SIRS varied from 99.5% (defined as having two or more criteria transiently present during a 24-hour period of automatic recording) to 66.4% (defined as having three or four criteria simultaneously present with manual recording at hourly intervals), and the incidence of sepsis ranged subsequently from 31.1% to 25.1% (RR = 0.81, 95% CI = 0.71 to 0.92). The PPV of having an infection was 31.2% and 37.7% for the respective settings, the NPV was 100% and 82.1%. In non-infected patients, 60.0% of patients had three or more SIRS criteria. The frequency of having two or more SIRS criteria varied from 79.2% in the first 2 hours of admission compared with 70.2% 12 to 24 hours after admission.

## Conclusions

The measured incidence of SIRS and sepsis heavily depended on minor variations in modes of data recording and interpretation of diagnostic criteria. A more precise definition of sepsis should be incorporated into the design of future clinical trials in sepsis in order to ensure the uniform recruitment of patients.
